# Female Reproductive Health - study of an egg donor population

**DOI:** 10.5935/1518-0557.20230046

**Published:** 2023

**Authors:** Tatiana Filipa Santos Oliveira, Márcia Barreiro, António Tomé, Emídio Vale-Fernandes

**Affiliations:** 1 ICBAS - School of Medicine and Biomedical Sciences, UMIB - Unit for Multidisciplinary Research in Biomedicine, University of Porto, Porto, Portugal; 2 Centro de Procriação Medicamente Assistida / Banco Público de Gâmetas, Centro Materno-Infantil do Norte Dr. Albino Aroso (CMIN), Centro Hospitalar Universitário de Santo António (CHUdSA), Porto, Portugal; 3 Departamento da Mulher e da Medicina Reprodutiva, Centro Materno-Infantil do Norte Dr. Albino Aroso (CMIN), Centro Hospitalar Universitário de Santo António (CHUdSA), Porto, Portugal; 4 ITR - Laboratory for Integrative and Translational Research in Population Health, University of Porto, Porto, Portugal

**Keywords:** egg donors, medically assisted procreation, public gamete bank, infertility, *in vitro* fertilization

## Abstract

**Objective:**

Infertility is one of the most significant reproductive health issues addressed with medically assisted procreation. This study looked into a potential correlation between the number of mature oocytes harvested and donor biological characteristics in order to propose an anti-Müllerian hormone (AMH) cutoff level to optimize the selection of candidates for gamete donation.

**Methods:**

The donors were healthy women included in the Public Gamete Bank between 2011 and 2021. Their results can be used as a national indicator of fertility.

**Results:**

We found that women with higher AMH levels had more antral follicles and oocytes harvested. As age increased, the number of oocytes harvested decreased. The suggested AMH cutoff level for successful donation was 1.12 ng/mL.

**Conclusions:**

The analysis of the reproductive health of Public Gamete Bank donors allows the standardization of AMH cutoff values at a national level, since the same laboratory techniques were employed consistently across medical centers. The study also allowed insight into the factors that compromise donation success. If adopted, a more rigorous selection of donor candidates would increase the success rate of egg donations.

## INTRODUCTION

Sexual health has been the subject of discussion for decades, with the first definition of the term published in 1975. According to the World Health Organization, sexual health involves the physical, emotional, mental and social components of individuals (Edwards & Coleman, 2004).

Infertility, a condition that affects 8-12% of couples worldwide, is established when there is no pregnancy after one year of frequent unprotected sexual intercourse. Couples can suffer from primary infertility, if they have never been able to get pregnant, or secondary infertility, when they cannot become pregnant after at least one previous failed or successful pregnancy. Infertility can be idiopathic or due to female, male or mixed causes. Smoking, drinking, and drug use have been related to fertility decline (Vander Borght & Wyns, 2018).

Regarding ovarian reserve markers, female age is inversely proportional to female fertility, which decreases significantly after 30 years of age (Crawford & Steiner, 2015; Jehan & Syed, 2016). Elevated body mass index (BMI) is another factor that negatively affects fertility (Jehan & Syed, 2016). Anti-Müllerian hormone (AMH) is considered by many authors as the best marker of ovarian reserve, since it shows an early decrease with age when compared to other markers, and presents smaller oscillations throughout the ovarian cycle (it can be measured at any time of the month) (Coccia & Rizzello, 2008). The progression of antral to dominant follicles is mediated by follicle stimulating hormone (FSH); therefore, a high level of this hormone may indicate a low ovarian reserve. Antral follicle count (AFC), which decreases with age, is directly proportional to ovarian reserve (Deadmond *et al*., 2022).

Treatments involving donor eggs may provide a solution to infertility and can be used by couples on medically assisted procreation (MAP) who are unable to conceive due to poor oocyte quality, premature ovarian insufficiency, or risk of transmitting a serious anomaly to their offspring. Female gametes can be used fresh or cryopreserved. Fresh eggs are considered the desired standard of treatment, although they have been associated with greater difficulty in synchronizing donors and receivers and treatment delays caused by temporary donor shortage. For these reasons, cryopreserved eggs offer greater convenience to MAP centers, which is why they have been increasingly used (Lindheim & Klock, 2018; Kushnir & Gleicher, 2016; Nagy *et al*., 2020).

In the specific case of in vitro fertilization with donor gametes, success rates have been on the rise, with assisted reproductive technology accounting for one to three percent of all pregnancies in the United States and Europe. Since the 1990s, GnRH antagonists have offered an advantage over GnRH agonists in that they lead to suppression of the pituitary gland more quickly with fewer subcutaneous injections required. However, GnRH antagonists can be rather expensive (Goldberg *et al*., 2007; Eskew & Jungheim, 2017).

In February 2011, approval was given for the creation of the Public Gamete Bank (PGB) in Portugal at Centro Hospitalar Universitário de Santo António (CHUdSA). Extended access to MAP for all couples and women, regardless of marital status, sexual orientation or diagnosis of infertility, as well as access to surrogacy, increased the need to create a national network of public centers for MAP affiliated to the PGB (Diário da República nº 8/2017; Banco Público de Gâmetas, 2020).

## MATERIAL AND METHODS

This study included egg donor candidates registered with the PGB from June 2011 to September 2021. The CHUdSA Ethics Review Committee approved the study design. Candidate data were anonymized.

Data collection using the PGB database included a series of parameters: age, nationality, BMI, profession, marital status, education, blood group (ABO system and Rh factor), genetic tests (karyotyping, cystic fibrosis, spinal muscular atrophy, hemoglobinopathies and fragile X syndrome), number of previous donations and pregnancies, number of live births, stillbirths, previous abortions (spontaneous or induced), smoking, drinking, drug or occupational habits, medical history (diseases, surgeries, chronic medication and risky behavior), family history (diseases linked to the X chromosome, Down syndrome, Klinefelter syndrome, Turner syndrome, trisomies 13 and 18, spina bifida, cleft lip, osteogenesis imperfecta, achondroplasia, Steinert muscular dystrophy, spinal muscular atrophy, cystic fibrosis, diabetes, congenital metabolic disease, hemochromatosis, 21-hydroxylase deficiency, β-thalassemia, sickle cell disease, factor V Leiden, neoplasia of the breast, ovary or colon, retinoblastoma, neurofibromatosis, simple or malignant myopia, mucopolysaccharidosis, polycystic kidney, phocomelia, congenital pyloric stenosis, congenital heart disease, mental retardation, early senility , infertility and immune deficit), neurological and psychiatric history (personal or family conditions such as bipolar disorder, epilepsy, suicide, Huntington’s chorea and schizophrenia), gynecological history (menarche, regular or irregular menstrual cycle, presence or absence of dysmenorrhea, onset of sexual activity and type of contraception), ovarian reserve markers (AMH level and AFC in pelvic ultrasound) and parameters of the cycle of controlled ovarian stimulation for donation (type and dose of gonadotropins, days of stimulation and number of oocytes obtained). Psychological assessment and reason for rejection of the donation, when present, were also analyzed.

The age of candidates ranged from 18 to 35 years. Individuals with sexually transmitted diseases or hereditary diseases are not accepted as egg donors, for which anonymity must be guaranteed (Banco Público de Gâmetas, 2020; Samorinha *et al*., 2020; Soares *et al*., 2023). Among other reasons, careful donor screening is performed to identify factors that exclude donors from MAP. Excluded individuals must present at least one of the factors described in Table 1.

**Table 1:** Exclusion criteria for candidate donors.

Unsatisfactory clinical history
BMI > 35 kg/m^2^
Absence of sexual activity
Negative evaluation of the psychology consultation
AFC < 5 and/or AMH ≤ 1 ng/ml
Genetic disease
Altered serologies
Altered thyroid and/or pituitary function
History of ovarian hyperstimulation syndrome in a previous stimulation cycle
Less than 6 developing follicles in stimulation cycle
Poor oocyte quality

Data were processed on software program SPSS (Statistical Package for the Social Sciences) version 27 for Windows. The analysis involved measures of descriptive statistics (absolute and relative frequencies, means and respective standard deviations) and inferential statistics. The significance level to reject the null hypothesis was set at (α) ≤ 0.05. Significance levels are presented as: *for *p*<0.05, **for *p*<0.01 and ***for *p*<0.001. Pearson’s correlation coefficient, the ROC curve, Spearman’s correlation coefficient, Student’s t-test for independent samples, the Mann-Whitney test, One-Way ANOVA and the Kruskal-Wallis test were used. Distribution normality was analyzed using the Kolmogorov-Smirnov test and homogeneity of variances using the Levene’s test.

## RESULTS

At first, 466 egg donor candidates were included in the study. Most were Caucasian (90.6%) with ages ranging between 18 and 35 years, with an average age of 30 years. Portuguese nationality as well as residence in the Porto district were more frequent. A large proportion of them (76.7%) were single at the time of application. Almost a third (32.6%) of the egg donors were university students.

The average BMI of the candidates was 23.67 kg/m^2^. Regarding the phenotype, more than 80% had brown hair and brown eyes, and more than half had straight hair.

A total of 344 candidates were excluded. The most frequent reasons were failure to attend follow-up appointments and giving up on egg donation. The present study focused on the remaining 122 women. The donors had a BMI (22.84 kg/m^2^) and phenotype similar to the total population considered initially.

The selected population underwent gynecological assessment and were asked questions about their obstetric, medical, psychiatric and family history. A small portion of the included donors had donated gametes previously (11.5% had made a previous donation and 1.6% had made two donations). Thirty-three women had a history of successful pregnancy and 75% of the women with a history of abortion (13.2%) had their pregnancies voluntarily terminated. Oral contraceptives and barrier methods, used simultaneously, were the most frequently used methods (48.6%). The mean age at menarche was 12 years with a standard deviation of ±1.78; the start of sexual activity occurred at 17 years of age with a standard deviation of ±2.08.

Possible risk factors that might compromise the donation of female gametes were also analyzed, namely smoking, drinking, drug use, exposure to chemical substances and exposure to radiation. Around 17.4% of the women smoked, with the majority (95.2%) smoking less than a pack a day. The variable “drinking” was divided into “yes,” corresponding to frequent drinking, and “no,” pertaining to no or sporadic drinking. None of the donors in the selected population was a heavy drinker. One candidate used hashish and cannabis sporadically and two candidates were exposed to chemical substances, namely acids, methanol and phenol. None had exposure to radiation.

The sample of selected candidates was submitted to controlled ovarian stimulation. In all cases, GnRH antagonists were used and all of them were given recombinant gonadotropins. It should be noted that the use of cryopreserved oocytes has increased over the years to the detriment of fresh oocytes (Table 2).

**Table 2:** Comparison between the number of cryopreserved and fresh oocytes used in the years from 2011 to 2021.

Year	Number of cryopreserved oocytes	Number of oocytes used fresh
2011	0	56
2012	0	25
2013	0	58
2014	13	42
2015	6	135
2016	17	57
2017	197	38
2018	261	0
2019	246	0
2020	89	0
2021	200	0

The AMH levels recorded in 75 women ranged between 1 and 8.59 ng/mL, with an average of 3.81 ng/mL. The relationship between AMH level and age, BMI, days of stimulation and total dose of gonadotropins, number and size of follicles obtained (< or ≥15mm), number of oocytes obtained and number of antral follicles in pelvic ultrasound, as well as their relationship with smoking, psychiatric, medical and family history, was analyzed. We were unable to carry out a statistical analysis of the association between AMH level and genetic test results or relevant donor diseases, or with risk factors, with the exception of smoking, since the percentage of changes in these parameters was negligible.

Significant correlations were found between AMH level and number of follicles ≥ 15mm, number of follicles < 15mm, number of antral follicles, and number of oocytes harvested. Since the correlation coefficients were positive, the higher the AMH level, the greater the number of follicles (regardless of size), antral follicles, and oocytes harvested. The correlation with total dose was negative, i.e., low AMH levels were associated with higher dosages administered in the stimulation protocol (Table 3). Candidates with a considerable psychiatric history had significantly lower AMH levels.

**Table 3:** Relationship of AMH level with age, BMI, days and stimulation dose, follicle size, number of antral follicles, and oocytes harvested.

	AMH
Age	-.144
BMI	-.145
Stimulation days	-.157
Total dose	-.341^**^
Number of follicles ≥ 15mm	.500^***^
Number of follicles < 15mm	.456^***^
Number of oocytes harvested	.517^***^
Number of antral follicles	.421^***^

* *p*<0.05

** *p*<0.01

*** *p*<0.001.

The same analysis was performed for number of oocytes. Statistical analysis of the relationship between number of oocytes and genetic test results, relevant donor diseases, risk factors, with the exception of smoking, and psychiatric history was not performed, since the percentage of alterations in these parameters was negligible.

Significant correlations were found between number of oocytes harvested and number of follicles regardless of size, and number of antral follicles. Since the correlation coefficients were positive, the higher the number of parameters analyzed, the greater the number of oocytes harvested. The correlation was negative with regard to age, i.e., as age increased, the number of oocytes harvested decreased (Table 4). Candidates with a considerable family history had a significantly higher number of oocytes harvested.

**Table 4:** Relation of the number of oocytes obtained with age, BMI, days and stimulation dose, follicle size and number of antral follicles.

	Number of oocytes obtained
Age	-.248^**^
BMI	.007
Stimulation days	-.195
Total dose	-.125
Number of follicles ≥ 15mm	.439^**^
Number of follicles < 15mm	.589^**^
Number of antral follicles	.198^*^

* *p*<0.05

** *p*<0.01

*** *p*<0.001.

We searched for an AMH cutoff level to select egg donor candidates from the initial sample of 466 women. The quantitative variable studied was the AMH level and the qualitative variable was completion of donation, in which “yes” corresponded to successful gamete donation, with 75 women, and “no” to having a low ovarian reserve, with 10 women. Table 5 shows a statistically significant (*p*<0.001) area under the ROC curve (0.961), which means the model had an excellent discriminative capacity. The suggested cutoff for the AMH level for successful completion of donation was 1.12 ng/mL.

**Table 5:** Relationship between AMH level and donation completion.

Test Result Variable(s): AMH				
**Area**	**Std. Error^a^**	**Asymptotic Sig.^b^**	**Asymptotic 95% Confidence Interval**	
			**Lower Bound**	**Upper Bound**
.961	.024	.000	.914	1.000

a Under the nonparametric assumption

b Null hypothesis: true area = 0.5

The 75 women with completed gamete donations were divided into two groups (under 30 years old and over 30 years old) in an attempt to find a potential association between AMH cutoff levels and age. However, the size of each group (38 women under 30 years old and 37 women over 30 years old) was not sufficient to carry out a statistical analysis for AMH cutoff level.

## DISCUSSION

This study included gamete donors enrolled with the PGB from 2011 to 2021 and allowed inferences about the fertility of the female population at a national level and the elements related to successful and failed egg donation.

It included 466 women, most of whom were Caucasian. More than three quarters (76.7%) were single and almost a third (32.6%) were university students. They were aged 30 years on average and the mean BMI was within the normal range, as per the donation criteria. More than 80% had brown hair and eyes, and more than 50% had straight hair, which denotes little heterogeneity in the sample. Most (405) were Portuguese nationals and 225 lived in the Porto district, which shows the local activity of centers affiliated with the PGB, limiting the extrapolation of this study to the national population.

Withdrawal from egg donation and failure to attend follow-up visits were the main reasons for the exclusion of egg donor candidates, which brought the number of included individuals down to 122 women. The reasons for dropping out must be analyzed in order to increase the number of donors.

Smoking, drinking, drug use, and exposure to chemical substances or radiation were some of the risk factors analyzed. However, the influence of these parameters on egg donation was not demonstrated, since the percentage of women who had any of these risk factors was negligible. Regarding the conclusions obtained by other authors, it should be noted that ovarian stimulation was quite reduced in smoking compared to non-smoking donors (Fréour *et al*., 2018). Another study associated smoking with decreased fertility in oocyte donors (Melnick & Rosenwaks, 2018). In the present study, 17.4% of the candidates smoked, although 95.2% smoked less than a pack a day. The results obtained by light smokers (less than 10 cigarettes per day) were equivalent to those of non-smokers; unfavorable outcomes were essentially observed in heavy smokers (Soares *et al*., 2007; Rockhill *et al*., 2019).

One donor used hashish and cannabis sporadically. However, the relationship between drug use and fertility is unclear (Har-Gil *et al*., 2021).

Although heavy drinking was not described in our population, alcoholism has been associated with reduced fertility. According to Silvestris *et al*. (2019), exposure to chemical substances and environmental pollutants may cause irreparable genetic mutations in gametes, a situation experienced by two candidates in this study.

Exposure to radiation, especially from cancer treatment, may compromise fertility, although no case was found in this study. Factors such as site of irradiation, dose and intensity, patient age, and use of combined chemotherapy increase the risk of developing reproductive impairment in the short or long term (Imai *et al*., 2008).

Cryopreserved oocytes have been more used than fresh eggs, as also seen in the present study, which reduces the cost of treatment (Kushnir & Gleicher, 2016).

AMH level, an ovarian reserve marker, was measured in 75 women. In our study, the higher the AMH level, the greater the number of follicles and oocytes. This has been confirmed in the literature, along with a positive correlation between AMH and AFC and a negative correlation between AMH level and FSH (Shrikhande *et al*., 2020; Kotanidis *et al*., 2016). In the statistical analysis of this study, no significant correlation was found between AMH level and donor age or BMI. However, several studies found that AMH levels decrease as age increases from the age of 25 years, and as the BMI increases (Kotanidis *et al*., 2016; Savas-Erdeve *et al*., 2017; Kotlyar & Seifer, 2021).

We also found that age was inversely related with the number of oocytes harvested, with aging associated with declining fertility (Vollenhoven & Hunt, 2018).

The lack of an international standard value for AMH levels makes it difficult to compare between studies. In addition, there are several laboratory kits to measure AMH, which leads to variability in the results between different medical centers (Moolhuijsen & Visser, 2020). The European Society of Human Reproduction and Embryology indicated that AMH levels below 0.5-1.1 ng/mL are associated with low ovarian reserve (Bedenk *et al*., 2020). In the present study, an AMH cutoff level of 1.12 ng/mL was suggested, with levels above the cutoff value being correlated with successful donation. Baker *et al*. (2021) predicted a cutoff level of 0.93 ng/mL. According to Vale-Fernandes et al. (2023), a threshold value of AMH = 3.2 ng/mL predictive of the retrieval <12 oocytes (areas under the curve, 0.7364; 95% confidence interval: 0.529-0.944) was identified by receiver operating characteristic curve. Using this cutoff, the normal response (12 oocytes) was predicted with a sensitivity of 77% and a specificity of 60%.

The lower the AMH level, the higher the dose to be administered in ovarian stimulation, a finding corroborated by Nakhuda *et al*. (2010).

The analysis of an association between psychiatric conditions and lower AMH levels and the presence of a considerable family history and higher number of oocytes harvested were inconclusive and not described in the literature.
